# IL-1β as an important mediator of macrophage–chondrocyte crosstalk in pararamosis (rainforest arthritis)

**DOI:** 10.3389/fimmu.2026.1880784

**Published:** 2026-08-25

**Authors:** Paula C. Pohl, Luiza N. K. Zapotoski, Luiz R. Sardinha, Isadora M. Villas-Boas, Giselle Pidde, Denise V. Tambourgi

**Affiliations:** Immunochemistry Laboratory, Butantan Institute, São Paulo, SP, Brazil

**Keywords:** chondrocytes, macrophages, osteoarthiritis, pararama, Premolis semirufa

## Abstract

**Background:**

Pararamosis, or pararama-associated phalangeal periarthritis, is a neglected tropical disease affecting rubber tappers in the Amazon, caused by contact with the urticating bristles of the *Premolis semirufa* caterpillar. This condition leads to chronic synovitis and progressive cartilage degradation, key features shared with other osteoarticular conditions such as osteoarthritis.

**Methods:**

We investigated the inflammatory mechanisms of pararamosis by examining macrophage polarization and the subsequent crosstalk with chondrocytes. THP-1-derived macrophages were treated with *P. semirufa* bristles extract (PsE) to assess polarization and inflammasome activation. Furthermore, we compared inflammatory and catabolic markers in chondrocyte monocultures *versus* macrophage-chondrocyte co-cultures.

**Results:**

PsE stimulation induced a robust M1 macrophage phenotype, evidenced by the upregulation of CD80, CD86, and TLR2, alongside increased expression of inflammasome components (*NLRP3* and *CASP1*) and pro-inflammatory cytokines (*IL1B, IL6, IL8, IL23A*, and *IL33*). These transcriptional changes were accompanied by increased IL-1β, IL-6, and TNF-α secretion, confirming the pro-inflammatory phenotype. While PsE directly induced chondrocytes to express matrix metalloproteinases (MMPs) and inflammatory cytokines, these effects were significantly amplified in co-culture. The macrophage-chondrocyte interaction exacerbated the secretion of IL-6, IL-11, IL-23, CCL2, CCL5, MMP-1, MMP-3, and MMP-13. Crucially, antibody-mediated neutralization of IL-1β in co-cultures significantly attenuated the production of IL-11, IL-23, MMP-1, and MMP-3, while other pathways likely contribute to the regulation of additional inflammatory mediators.

**Conclusion:**

Our findings demonstrate that PsE promotes a sustained pro-inflammatory macrophage response and establishes a pathogenic crosstalk between macrophages and chondrocytes that amplifies inflammatory and catabolic signaling. The data identify IL-1β as a major regulator of this interaction and support the involvement of inflammasome-related pathways in the response to PsE. These results advance our understanding of the mechanisms underlying pararamosis and reveal biological features shared with osteoarthritis, providing a foundation for future studies aimed at validating the role of IL-1β and other inflammatory mediators *in vivo*.

## 1 Introduction

Envenomation by caterpillars of *Premolis semirufa* constitutes a neglected yet clinically relevant inflammatory condition that primarily affects forest workers in the Amazon region (1, 2). The condition, referred to as Pararamosis, results from accidental contact with caterpillar bristles and evolves from an acute urticarial reaction to a progressive chronic phase characterized by persistent periarticular inflammation. Recurrent envenomation is strongly associated with synovial membrane thickening, periarticular fibrosis, cartilage damage, and irreversible deformities of the interphalangeal joints, ultimately leading to functional impairment (1, 3, 4). Radiological findings from affected individuals reveal soft tissue swelling, joint space narrowing, and skeletal alterations resembling those observed in degenerative joint diseases such as osteoarthritis (5).

The toxic activity of the bristles is attributed to secretions produced by specialized trichogen cells located at their base, which release bioactive compounds including proteolytic enzymes and proinflammatory molecules. These components penetrate the skin and trigger a robust local inflammatory response (6). The chronic and progressive osteoarticular manifestations of Pararamosis suggest that, beyond mechanical injury, sustained immunological and inflammatory mechanisms play a central role in disease pathogenesis.

Despite its social and economic impact on affected populations, Pararamosis remains largely overlooked in public health policies, and the molecular and cellular mechanisms underlying joint damage remain poorly understood. Previous experimental studies have demonstrated that extract from *P. semirufa* bristles (PsE) induces a marked joint inflammatory response *in vivo*, characterized by prominent macrophage and neutrophil infiltration, as well as T lymphocyte and antigen-presenting cell activation (7). *In vitro* studies also reveal that PsE directly activates neutrophils, enhancing pro-inflammatory mediator release and degranulation (8). Subsequent investigations showed that PsE directly activates the human complement system - generating the anaphylatoxins C3a, C4a, and C5a - and contains a serine protease with significant proteolytic activity, thereby amplifying inflammation and tissue damage (9, 10). Supporting these findings, multi-omics analyses identified PsE proteins homologous to human molecules involved in chemotaxis, cell adhesion, apoptosis, cytoskeletal dynamics, and extracellular matrix (ECM) remodeling, processes critically implicated in joint degeneration (11).

Beyond its effects on immune cells, PsE also disrupts cartilage homeostasis. Functional assays using human chondrocytes demonstrated that exposure to the extract induces the release of inflammatory mediators associated with osteoarthritis pathogenesis, including IL-6, CXCL8, CCL2, prostaglandin E2, complement components, and matrix metalloproteinases (MMP-1, MMP-2, MMP-3, and MMP-13). Consistently, transcriptomic analyses revealed the activation of inflammatory signaling pathways, immune cell chemotaxis programs, and ECM remodeling signatures, establishing an osteoarthritis-like molecular profile in treated chondrocytes (12).

More recently, the direct effects of PsE on synovial cells have been investigated. Studies using human synoviocytes and macrophages demonstrated that PsE induces a pronounced proinflammatory phenotype, characterized by increased production of cytokines, chemokines, and matrix-degrading enzymes (13).

Macrophages are central orchestrators of joint inflammation and tissue remodeling. In osteoarthritis and other chronic arthropathies, synovial macrophage activation drives the production of proinflammatory cytokines such as IL-1β, TNF-α, and IL-6 (14, 15). These mediators act on chondrocytes—the sole resident cells of articular cartilage—inducing the expression of matrix metalloproteinases and aggrecanases that degrade ECM components. In turn, activated chondrocytes release additional cytokines, chemokines, and growth factors, reinforcing macrophage activation and establishing a self-perpetuating cycle of synovial inflammation and cartilage destruction (16–18).

Despite advances in Pararamosis research, the direct effects of *P. semirufa* on resident and infiltrating joint cells and, critically, the mechanisms governing macrophage-chondrocyte interactions remain insufficiently characterized. Given the pivotal role of macrophages in orchestrating synovial inflammation and of chondrocytes in maintaining cartilage integrity, elucidating the crosstalk between these cell types is essential for understanding the pathophysiology of joint damage induced by *P. semirufa*.

Here, we investigated the inflammatory mechanisms underlying Pararamosis, focusing on macrophage-chondrocyte crosstalk. Using a co-culture system comprising human THP-1-derived macrophages and primary human articular chondrocytes, we evaluated macrophage polarization profiles, inflammasome activation, and downstream effects on chondrocyte inflammatory and catabolic responses following PsE stimulation. Additionally, we assessed the impact of IL-1β neutralization on cytokine and metalloproteinase production to determine its role in mediating macrophage-chondrocyte communication. Our findings provide novel insights into the immunopathology of Pararamosis and uncover molecular links between environmental inflammatory triggers and degenerative joint diseases such as osteoarthritis.

## 2 Materials and Methods

### 2.1 *P. semirufa* bristles extract (PsE) preparation

Caterpillars of *P. semirufa* were collected in São Francisco, Pará, Brazil, under authorization from ICMBIO (protocol no. 11971-2). Access to biological material was approved by IBAMA (process no. 02001.008743/2011-12, authorization no. 01/2009) and SisGen (registration A05C092, 11/05/2018). Bristles were excised, suspended in phosphate-buffered saline (PBS; pH 7.4) and stored at −80 °C in Immunochemistry Laboratory at the Butantan Institute.

For *P. semirufa* bristles extract (PsE) preparation, samples were macerated using a glass rod, centrifuged to remove insoluble material, passed through a 0.22 µm membrane filter (Whatman-GE Healthcare, Chicago, IL, USA), aliquoted, and stored at −80 °C until use. Total protein content was quantified using a bicinchoninic acid (BCA) assay (Pierce Biotechnology, Thermo Fisher Scientific Inc., Waltham, MA, USA), according to the manufacturer’s protocol.

To assess the presence of bacterial endotoxins (LPS), PsE samples were analyzed using the Pyrogent Turbidimetric TM-5000 LAL (Limulus Amebocyte Lysate) assay (Charles River Laboratories, Wilmington, MA, USA). The analysis was performed by the Microbiological Quality Control Sector at the Butantan Institute, following standard operating procedures. Results were expressed in endotoxin units per milliliter (EU/mL).

### 2.2 THP-1 cell culture and macrophage differentiation

THP-1 human monocytic cells were obtained from the Rio de Janeiro Cell Bank (BCRJ, Rio de Janeiro, Brazil) and maintained at a density of 0.2 to 1.0 x 10^6^ cells/mL in RPMI 1640 medium (Gibco, Grand Island, NY, USA) supplemented with 25 mM glucose, 10 mM HEPES, 1 mM sodium pyruvate, 24 mM sodium bicarbonate, 1% penicillin–streptomycin, and 10% fetal bovine serum (FBS). Cultures were incubated at 37 °C in a humidified atmosphere containing 5% CO_2_. Cell viability and total cell number were routinely assessed by Trypan blue exclusion (0.4%) using a Neubauer hemocytometer.

For the experiments, THP-1 cells were seeded at a density of 1.65 x 10^5^ cells/well into 24-well plates (Costar^®^, Corning Inc., NY, USA) and differentiated into macrophages by treatment with 50 nM phorbol 12-myristate 13-acetate (PMA; Sigma–Aldrich, St. Louis, MO, USA) for 48 hours. Following differentiation, PMA-containing medium was removed and replaced with fresh supplemented RPMI, and cells were incubated for an additional 24 hours, being ready for the experiments.

### 2.3 Chondrocytes culture

Normal human articular primary chondrocytes (NHAC-Kn) were obtained from Lonza (Basel, Switzerland). The cells were cultured up to the 3rd passage in chondrogenic growth medium supplemented with growth factors (Lonza, Basel, Switzerland) at 37 °C and 5% CO_2_. Subsequently, the medium was gradually re-placed with DMEM/F12 medium (Sigma-Aldrich, St. Louis, MO, USA) supplemented with 14 mM sodium bicarbonate, 1% Penicillin-streptomycin solution, and 10% FBS, reaching full transition by the 5th passage and being ready for the experiments. Cell viability and total cell number were routinely assessed by Trypan blue exclusion (0.4%) using a Neubauer hemocytometer.

### 2.4 Treatment of THP-1-differentiated macrophages with PsE

THP-1 cells differentiated into macrophages according to item 2.2 were incubated with PsE (60 µg/mL) or PBS (equivalent volume to the extract) in RPMI medium supplemented with 1% FBS at 37 °C and 5% CO_2_. After 48 hours of treatment, cells were processed for RNA extraction or flow cytometry analysis.

### 2.5 Macrophage-chondrocyte coculture and PsE stimulation

For coculture assays, a transwell system was employed. Three days prior to stimulation, THP-1 cells were seeded at a density of 2.8 x 10^4^ cells *per* insert in 96-well transwell plates equipped with 0.4 µm pore membranes (Costar^®^, Corning Inc., NY, USA) and differentiated into macrophages using PMA, as previously described. In parallel, chondrocytes were plated at 1.2 x 10^5^ cells per well in 24-well plates (Costar^®^, Corning Inc., NY, USA) 24 h before stimulation. Coculture was initiated by transferring half of the transwell inserts containing differentiated macrophages into wells seeded with chondrocytes, while the remaining inserts and chondrocytes were maintained as monoculture controls. Cells were exposed to PsE (60 µg/mL) or PBS (vehicle control) in DMEM/F12 supplemented with 1% FBS, in a final volume of 700 µL. After 24 and 48 h of incubation, culture supernatants were collected from all cultures and stored at -80 °C for subsequent analyses, and RNA was extracted from chondrocytes cultured in mono- or coculture conditions. Cell viability was concurrently evaluated using the MTT assay.

### 2.6 IL-1β neutralizing experiment

Chondrocytes and THP-1 derived macrophages were cocultured as described above. Cells were pretreated with 5 µg/mL of anti-IL-1β antibody (MAB201, R&D Systems, Minneapolis, MN, USA) or IgG1 isotype control (MAB002, R&D Systems, Minneapolis, MN, USA) for 1 hour in DMEM/F12 supplemented with 1% FBS. Subsequently, PsE (60 µg/mL) or PBS (vehicle control) were added to the cocultured cells. After 48 hours of incubation, culture supernatants were collected and stored at -80 °C for further analysis and cell viability was assessed using the MTT assay.

### 2.7 Cell viability assessment (MTT assay)

Cell viability was determined using the MTT reduction assay (19). Following treatment with or without PsE, the supernatant was removed, and cells were incubated with MTT solution (0.5 mg/mL in DMEM/F12) for 3 h at 37 °C under 5% CO_2_. The resulting formazan crystals were dissolved in Dimethyl Sulfoxide (DMSO, Merck, Darmstadt, Germany), homogenized by gentle shaking for 2 min, and absorbance was measured at 540 nm using a spectrophotometer (Cytation 3 Cell Imaging Multi-Mode, BioTek, Winooski, VT, USA).

### 2.8 RT-qPCR

Total RNA was extracted from treated cells using TRIzol™ reagent (Invitrogen, Thermo Fisher Scientific Inc., Waltham, MA, USA) according to manufacturer’s protocol. RNA concentration and purity were determined at 260 nm and by the 260/280 nm ratio (≥1.8 considered acceptable), respectively, using a NanoDrop One spectrophotometer (Thermo Scientific, Thermo Fisher Scientific Inc., Waltham, MA, USA). To eliminate genomic DNA contamination, RNA samples were treated with DNase I (Invitrogen, Thermo Fisher Scientific Inc., Waltham, MA, USA) according to the manufacturer’s instructions. Reverse transcription was then carried out using 1 µg of DNase-treated RNA and SuperScript™ III Reverse Transcriptase kit (Invitrogen, Thermo Fisher Scientific Inc., Waltham, MA, USA), following the manufacturer’s protocol. Standard curves, relating cDNA dilution to cycle threshold (Ct) values, were used to calculate amplification efficiency, which was determined using QuantStudio™ Design & Analysis Software, version 1.5.1 (Applied Biosystems, Thermo Fisher Scientific Inc., Waltham, MA, USA). Primers with amplification efficiency between 90% and 110% were considered acceptable.

Quantitative real-time PCR was performed using a 10-fold dilution of cDNA from each biological sample, 250 nM of each primer (forward and reverse), and Fast SYBR™ Green Master Mix (Applied Biosystems, Thermo Fisher Scientific Inc., Waltham, MA, USA). A melting curve analysis was conducted at the end of each run to confirm the specificity of product amplification. Transcription levels were normalized to *GAPDH* and *PPIA* for macrophages-derived samples and *GAPDH* and *RPL13A* for chondrocytes-derived samples. The results were expressed as relative abundance using the 2^–ΔΔCt^ method (20). A complete list of primers used is provided in **Supplementary Table 1**.

### 2.9 Flow cytometry analysis

THP-1 derived macrophages obtained as described in Section 2.4 were detached using TrypLE™ Express Enzyme (Gibco, Thermo Fisher Scientific Inc., Waltham, MA, USA) and aliquoted at 1.5 x 10^5^ cells per tube (50 µL) in quadruplicates. Cells were washed twice with FACS buffer (1X PBS supplemented with 1% BSA and 0.05% sodium azide). Prior to staining, cells were incubated with 0.5 µg of human FcBlock (BD Biosciences, San Jose, CA, USA) per tube for 10 min to block Fc receptor-mediated non-specific binding. Cells were then incubated with the corresponding fluorescently conjugated antibodies at a 1:50 dilution for 30 minutes in the dark. After staining, cells were washed twice, resuspended in staining buffer (1X PBS supplemented with 1% BSA, 0.05% sodium azide and 1% paraformaldehyde), and kept at 4 °C until acquisition. Single-stained compensation controls were prepared using compensation beads (BD Biosciences, San Jose, CA, USA, 552843) according to the manufacturer’s instructions and stained individually with each fluorochrome-conjugated antibody used in the panel. Compensation matrices were calculated using these controls and applied to all samples during data analysis. Samples were acquired on a FACSLyric™ flow cytometer (Becton Dickinson, San Jose, CA, USA), and data was analyzed using FlowJo software v. 10.10.0 (BD Biosciences, San Jose, CA, USA). Cell populations were defined based on forward and side scatter parameters and marker expression was quantified as median fluorescence intensity (MFI). The following antibodies were used (all from BD Biosciences, San Jose, CA, USA): FITC anti- CD14 (555397), BV421 anti-CD80 (751723), PE anti-CD86 (555665), PerCP-Cy™5.5 anti- CD163 (563887), APC anti-CD206 (550889), PE anti-TLR2 (565349) e PE anti-TLR4 (564215).

### 2.10 Cytometric bead array (CBA) analysis

The concentrations of cytokines and chemokines in the supernatants of the cultures were quantified by flow cytometry using the following kits: Human Inflammatory Cytokine Cytometric Bead Array (551811) and Human Chemokine Kit (552990) according to the manufacturer’s recommendations (BD Biosciences, CA, USA). The samples were evaluated for the presence of the cytokines IL-1β, IL-6, IL-10, IL-12p70 and TNF-α and for the chemokines CCL2/MCP-1, CCL5/RANTES, CXCL8/IL-8, CXCL9/MIG, and CXCL10/IP-10. The concentration of each factor was determined using CBA analysis software v1.1.15 (BD Biosciences, San Jose, CA, USA).

### 2.11 ELISA

The concentrations of IL-11, IL-23A, MMP-1, MMP-3, and MMP-13 in cell culture mediums were quantified using the following commercial kits according to the manufacturer’s recommendations: Human IL-11 ELISA Kit (EHIL11, Invitrogen Inc, Thermo scientific, Waltham, MA, USA), Human IL-23 Uncoated ELISA Kit (88-7237-88, Invitrogen Inc, Thermo scientific, Waltham, MA, USA), Human MMP1 ELISA Kit (ab215083, Abcam, Cambridge, UK), Human MMP3 Elisa Kit (AB100607, Abcam, Cambridge, UK) and Human MMP13 ELISA kit (AB100605, Abcam, Cambridge, UK).

### 2.12 Statistical analysis

Statistical analyses were performed using GraphPad Prism version 8.4.3 (GraphPad Software, San Diego, CA, USA). Relative expression values and MFI were converted to log2 fold change (Log_2_FC) relative to the corresponding control of each independent experiment before statistical analysis. Data distribution was assessed using the Shapiro-Wilk test. For analyses performed within individual culture systems, parametric data were evaluated using unpaired t-test (for two groups) or one-way ANOVA (for multiple groups), followed by Tukey’s *post hoc* test for multiple comparisons. When data did not meet parametric assumptions, Mann-Whitney test (for two groups) or Kruskal-Wallis test (for multiple groups) followed by Dunn’s *post hoc* test was applied. For comparisons across different culture systems, data were log10-transformed to improve normality and homogeneity of variance and analyzed using two-way ANOVA considering culture type and experimental condition as independent factors, followed by Tukey’s multiple comparisons test. Differences were considered statistically significant when p-values met the following thresholds: *p ≤ 0.05, **p ≤ 0.01, ***p ≤ 0.001, and ****p ≤ 0.0001.

## 3 Results

### 3.1 *P. semirufa* bristles extract (PsE) induces a pro-inflammatory profile in THP-1 derived macrophages

**Figure 1:**
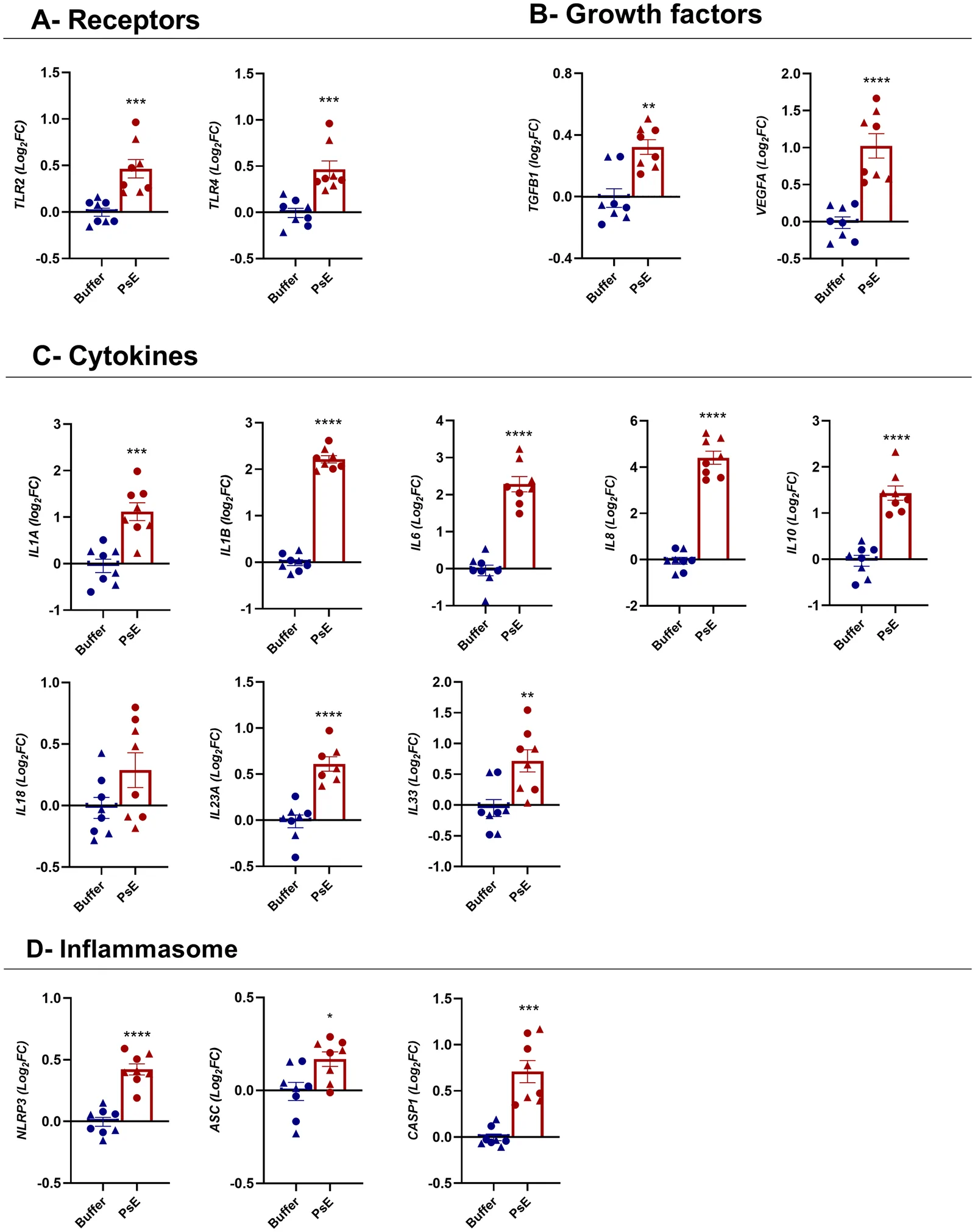
PsE induces inflammatory, growth factor, and inflammasome-related gene expression in macrophages. THP-1 derived macrophages were treated with *P. semirufa* bristle extract (PsE, 60 µg/mL) or buffer for 48 h. Relative mRNA expression of **(A)** receptors (*TLR2* and *TLR4*), **(B)** growth factors (*TGFB1* and *VEGFA*), **(C)** cytokines (*IL1A, IL1B, IL6, IL8, IL10, IL18, IL23A*, and *IL33*), and **(D)** inflammasome-related genes (*NLRP3, ASC*, and *CASP1*) was quantified by RT–qPCR. Expression levels were normalized to *GAPDH* and *PPIA* and expressed relative to the control (buffer). Data are presented as dot plots showing the mean ± SEM of the log_2_ fold change (log2FC) in relative gene expression from two independent experiments. Each data point represents an individual biological replicate, and symbol shape identifies the independent experiment (circles, Experiment A; triangles, Experiment B). Data were assessed for normality prior to statistical analysis and statistical significance was determined using Student’s *t*-test or Mann-Whitney test (*p ≤ 0.05, *p* ≤ 0.01, *p* ≤ 0.001 and ***p ≤ 0.0001).

Macrophages, the most abundant immune cell type in synovium, have critical importance in the symptomatology and progression of articular diseases. Considering that, we analyzed the effects of PsE on this cell type 48 h after incubation. Treatment of THP-1 derived macrophages with PsE markedly modulated the expression of genes associated with innate immune activation and response when compared to buffer-treated controls. PsE treatment resulted in a significant increase in the relative mRNA expression of *TLR2* and *TLR4*, indicating activation of pattern-recognition pathways involved in innate immune sensing (**Figure 1A**). In parallel, macrophages exposed to PsE showed an upregulation of growth factors, including *TGFB1* and *VEGFA* (**Figure 1B**). Gene expression analysis also revealed a robust induction of inflammatory cytokines following PsE treatment (**Figure 1C**). PsE-treated macrophages displayed increased mRNA levels of *IL1A*, *IL1B*, *IL6*, *IL8*, *IL10*, *IL23A*, and *IL33* when compared to controls. No statistically significant difference was observed in *IL18* expression between groups. Analysis of inflammasome-associated genes showed a significant increase in *NLRP3* and *CASP1* mRNA expression in PSE-treated macrophages relative to buffer-treated cells, whereas *ASC* exhibited a more modest modulation between groups (**Figure 1D**).

**Figure 2:**
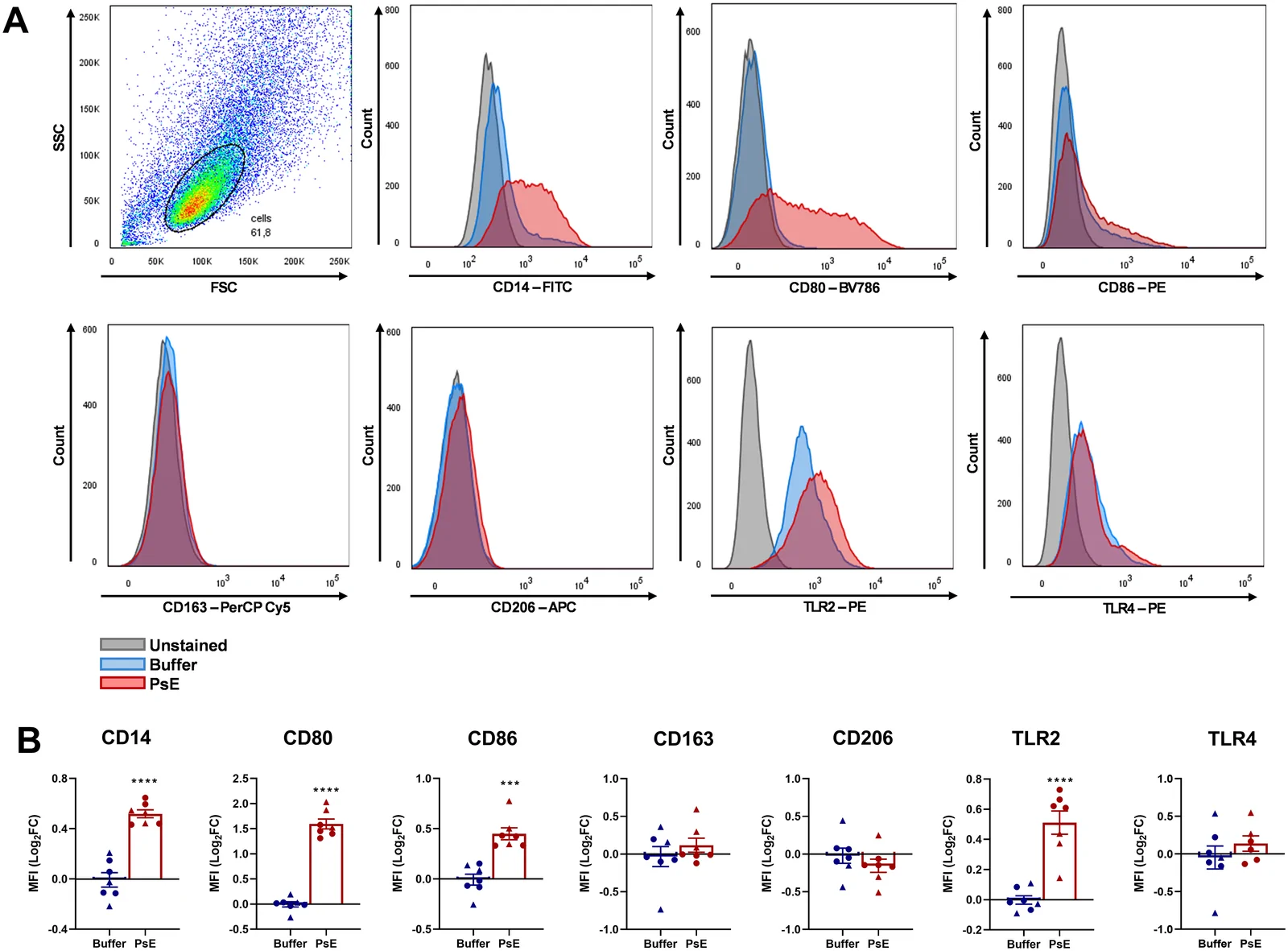
PsE modulates surface marker expression in macrophages. THP-1 derived macrophages were treated with *P. semirufa* bristle extract (PsE, 60 µg/mL) or buffer for 48 h, and the expression of CD14, CD80, CD86, CD163, CD206, TLR2, and TLR4 was analyzed by flow cytometry. **(A)** Representative gating strategy. Cells were initially selected based on forward scatter (FSC) and side scatter (SSC) to exclude debris and identify the main population. Marker expression was defined according to fluorescence intensity thresholds. Representative histograms illustrate marker expression in control (gray), untreated (buffer, blue), and PsE-stimulated (red) cells. **(B)** Quantification of surface marker expression. Data are presented as dot plots showing the mean ± SEM of the log_2_ fold change (log2FC) in the median fluorescence intensity (MFI) from two independent experiments. Each data point represents an individual biological replicate, and symbol shape identifies the independent experiment (circles, Experiment A; triangles, Experiment B). Data were assessed for normality prior to statistical analysis and statistical significance was determined using Student’s *t*-test or the Mann-Whitney test (***p ≤ 0.001 and ***p ≤ 0.0001).

Flow cytometric analysis further confirmed macrophage activation at the protein level. Representative flow cytometry plots illustrating the gating strategy are shown in **Figure 2A**. Cells were initially gated based on forward scatter (FSC) and side scatter (SSC) parameters to exclude debris and select the macrophage population. Subsequently, single-cell events were selected, and the expression of surface markers was determined based on fluorescence intensity using unstained and isotype controls to define positive populations. PsE treatment significantly increased the mean fluorescence intensity (MFI) of CD14, CD80, CD86, and TLR2, whereas TLR4 MFI remained unchanged. Expression of the alternative macrophage markers CD163 and CD206 remained unchanged (**Figure 2B**).

Endotoxin levels in the PsE were also assessed. The extract exhibited low endotoxin content, remaining below the detection limit of the assay. This result reinforced that the inflammatory responses observed in the experimental models are primarily driven by components of the PsE themselves.

### 3.2 Coculture with macrophages amplifies PsE-induced gene expression in chondrocytes

**Figure 3:**
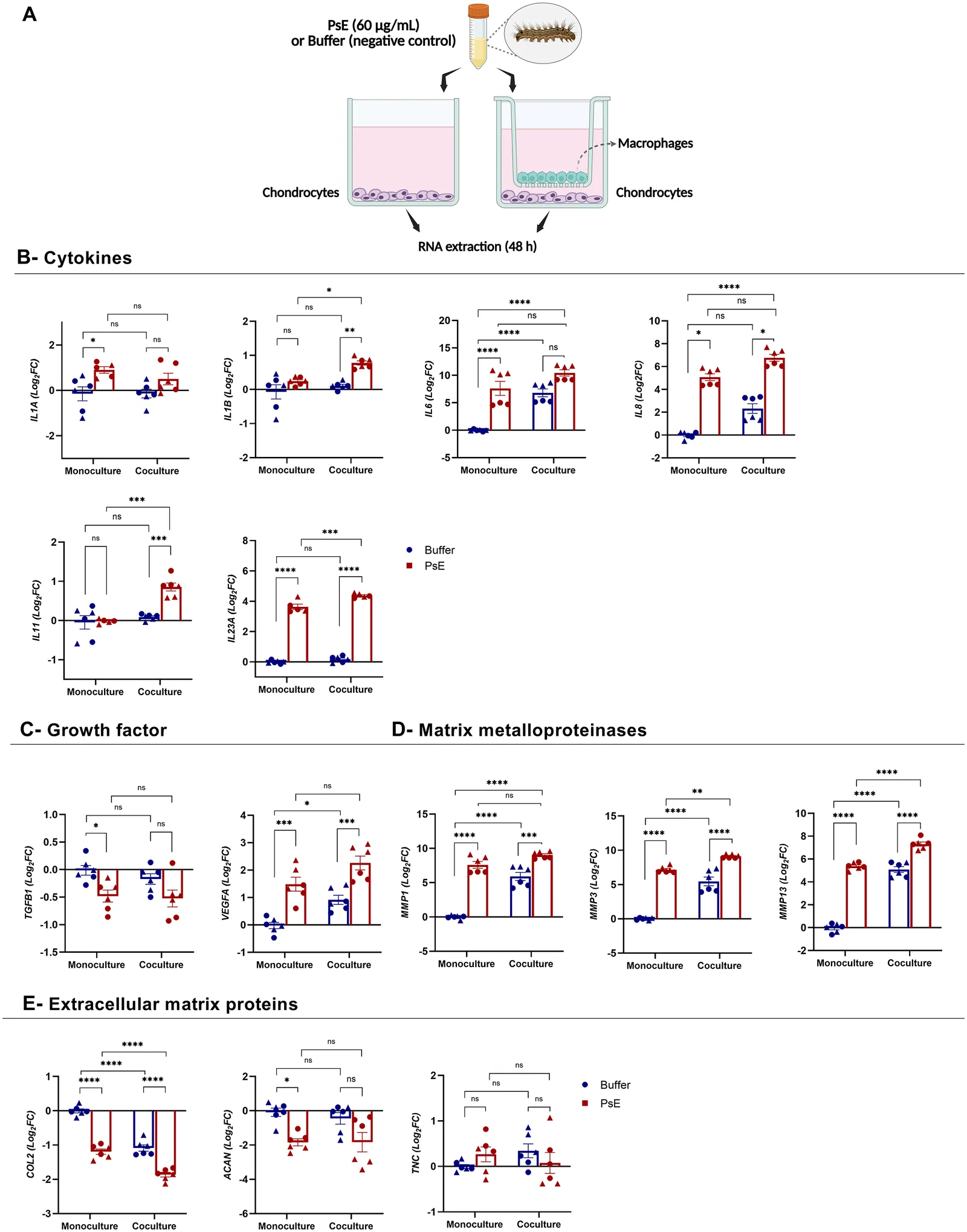
Macrophage–chondrocyte crosstalk amplifies PsE-induced gene expression in chondrocytes. Chondrocytes in monoculture or in coculture with THP-1 derived macrophages were treated with *P. semirufa* bristle extract (PsE, 60 µg/mL) or buffer for 48 h **(A)**. Relative mRNA expression of **(B)** cytokines (*IL1A, IL1B, IL6, IL8, IL11* and *IL23A*), **(C)** growth factors (*TGFB1* and *VEGFA*), **(D)** matrix metalloproteinases (*MMP1, MMP3* and *MMP13*) and **(E)** extracellular matrix proteins (*COL2, ACAN* and *TNC*) in chondrocytes was quantified by RT–qPCR. Expression levels were normalized to *GAPDH* and *PPIA* and expressed relative to the control (buffer). Data are presented as dot plots showing the mean ± SEM of the log_2_ fold change (log2FC) in relative gene expression from two independent experiments. Each data point represents an individual biological replicate, and symbol shape identifies the independent experiment (circles, Experiment A; triangles, Experiment B). Data were assessed for normality prior to statistical analysis and statistical significance was determined using one-way ANOVA followed by Tukey's test for parametric data, or Kruskal–Wallis followed by Dunn’s *post hoc* test for nonparametric data (*p ≤ 0.05, p ≤ 0.01, p ≤ 0.001 and *** p ≤ 0.0001). “ns” indicates not significant.

Interactions between chondrocytes and macrophages are key components of the joint microenvironment. To investigate this question, chondrocytes were cultured either in monoculture or in coculture with THP-1 derived macrophages and treated with PsE or buffer control. Gene expression was evaluated after 48 h of stimulation (**Figure 3A**). For several cytokines, significant differences were observed between monoculture and coculture conditions under PsE treatment, indicating a distinct expression profile associated with coculture. Under monoculture conditions, chondrocytes exhibited modest increased expression of the inflammatory mediators *IL6* and *IL8*. In contrast, these changes were more pronounced under coculture conditions with macrophages, in which chondrocytes treated with PsE showed increased expression of additional cytokines, including *IL1B*, *IL11*, *IL23A*, as well as higher transcript levels of *IL6* and *IL8* (**Figure 3B**).

Analysis of growth factor genes showed that *TGFB1* expression was slightly downregulated in PsE-treated monocultures, whereas no significant differences were observed in cocultures. However, PsE treatment significantly increased *VEGFA* expression in chondrocytes in both monocultured and cocultured chondrocytes (**Figure 3C**). The expression of matrix metalloproteinases was markedly affected by coculture conditions. While PsE treatment increased *MMP1*, *MMP3*, and *MMP13* expression in monocultured chondrocytes compared to buffer controls, substantially higher expression levels were observed when chondrocytes were cocultured with THP-1 macrophages and treated with PsE (**Figure 3D**). These increased expression of inflammatory and catabolic related genes was accompanied by reduced expression of extracellular matrix-related genes (*COL2A* and *ACAN*). Expression of *TNC* did not show significant differences between buffer- and PsE-treated groups under either culture condition (**Figure 3E**).

### 3.3 Coculture with macrophages enhances inflammatory and matrix-degrading mediator release

**Figure 4:**
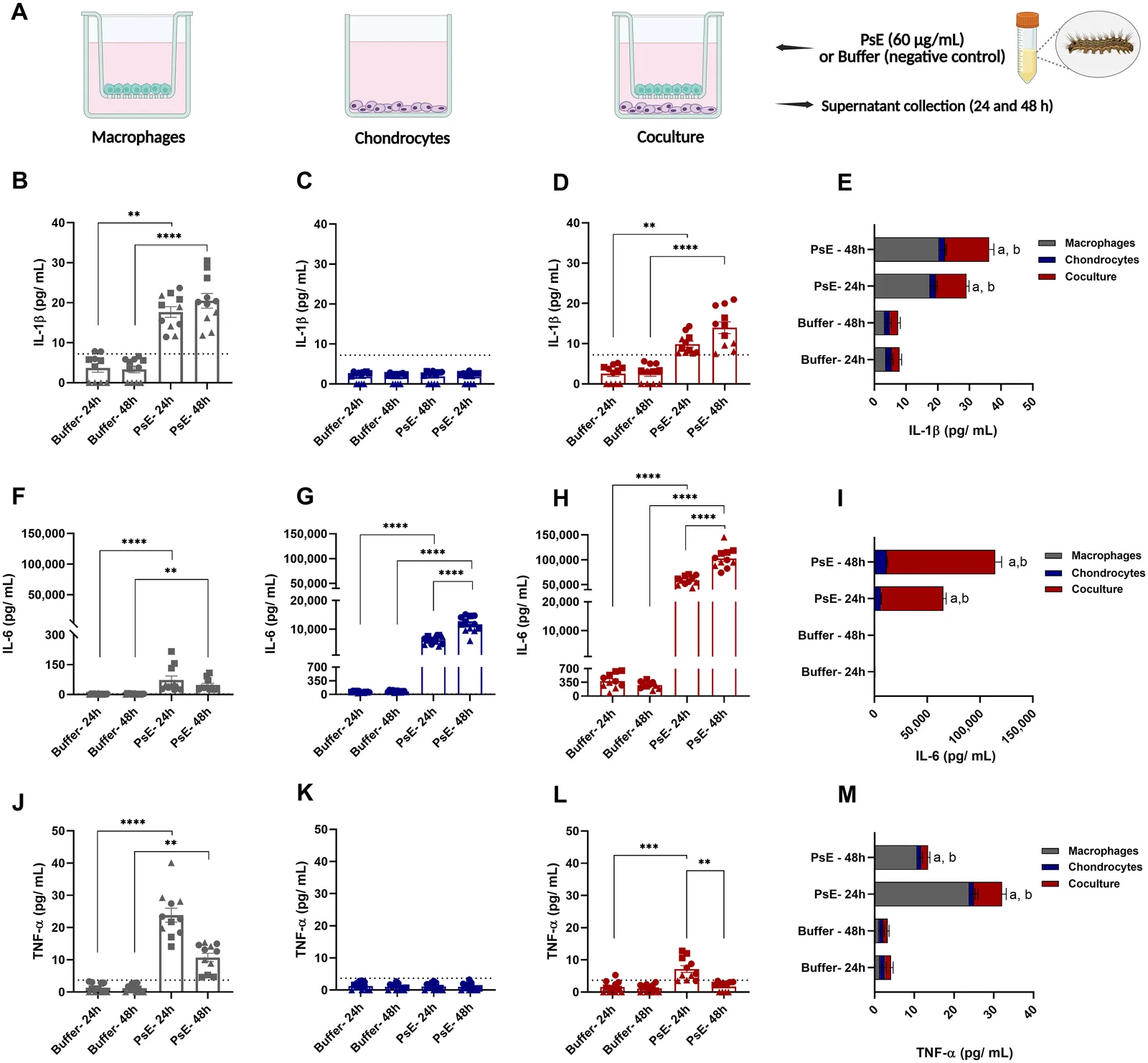
PsE modulates cytokine release in macrophage and chondrocyte cultures. THP-1 derived macrophages, chondrocytes, and macrophage–chondrocyte cocultures were stimulated with *P. semirufa* bristle extract (PsE; 60 µg/mL) or buffer (control) for 24 and 48 h **(A)**. The concentrations of IL-1β **(B–E)**, IL-6 **(F–I)**, and TNF-α **(J–M)** in culture supernatants were quantified by cytometric bead array (CBA). Data are presented as dot plots showing the mean ± SEM of biological replicates from three independent experiments. Each data point represents an individual biological replicate, and symbol shape identifies the independent experiment (circles, Experiment A; triangles, Experiment B; squares, Experiment C). Data were assessed for normality prior to statistical analysis. Statistical analyses were performed within each culture system using one-way ANOVA followed by Tukey’s *post hoc* test for parametric data, or Kruskal–Wallis followed by Dunn’s *post hoc* test for nonparametric data (** p ≤ 0.01, *** p ≤ 0.001 and **** p ≤ 0.0001). For comparisons between culture systems, data were log-transformed to meet parametric assumptions and analyzed using two-way ANOVA, where “a” indicates statistically significant differences between cocultures and macrophage monocultures, and “b” indicates differences between cocultures and chondrocyte monocultures. The dotted line represents the limit of detection for each analyte.

**Figure 5:**
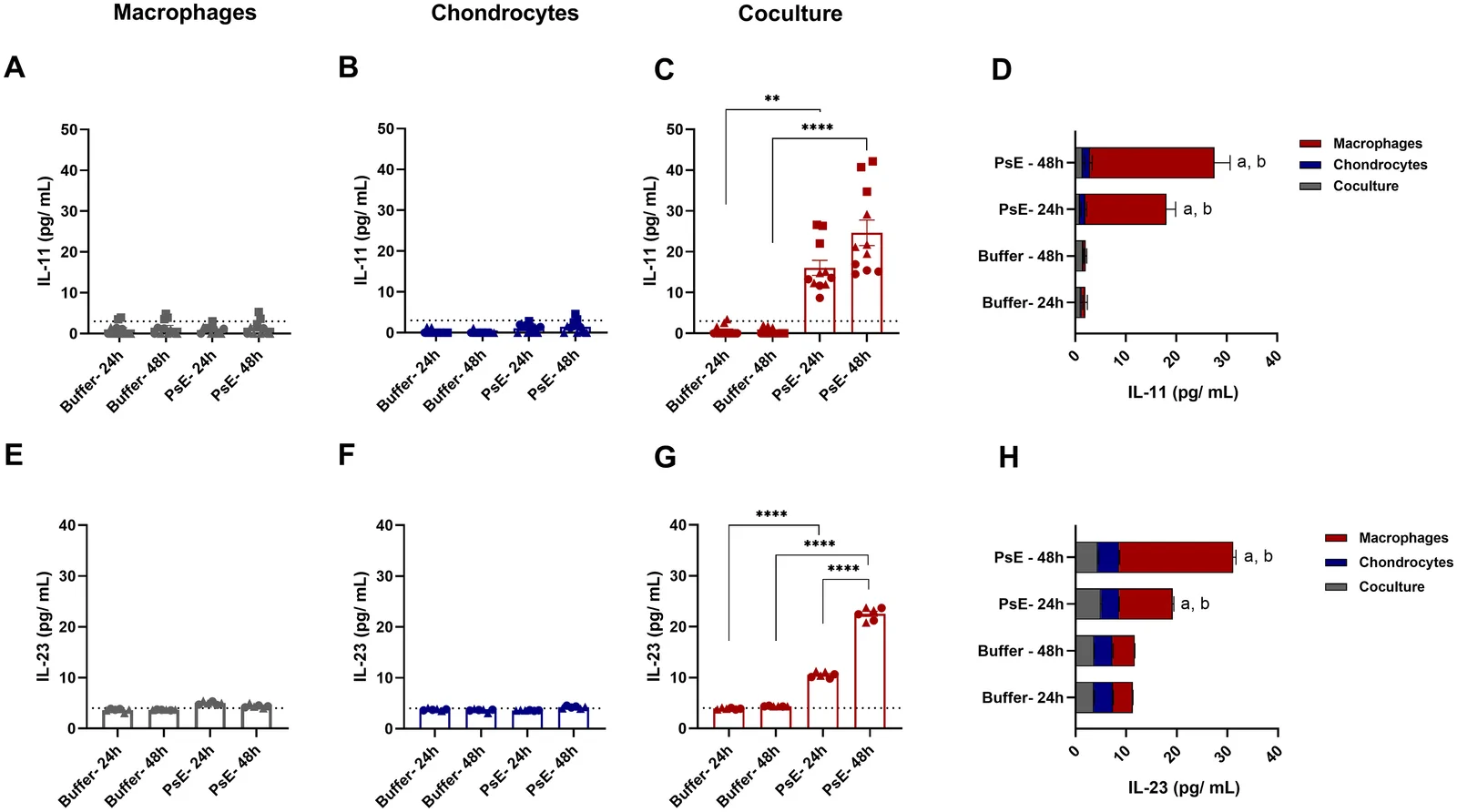
Macrophage–chondrocyte crosstalk induces the production of IL-11 and IL-23 in PsE-stimulated cultures. THP-1–derived macrophages, chondrocytes, and macrophage–chondrocyte cocultures were stimulated with *P. semirufa* bristle extract (PsE; 60 µg/mL) or buffer (control) for 24 and 48 h. The concentrations of IL-11 **(A-D)** and IL-23 **(E-H)** in culture supernatants were quantified by ELISA. Data are presented as dot plots showing the mean ± SEM of biological replicates from two or three independent experiments. Each data point represents an individual biological replicate, and symbol shape identifies the independent experiment (circles, Experiment A; triangles, Experiment B; squares, Experiment C). Data were assessed for normality prior to statistical analysis. Statistical analyses were performed within each culture system using one-way ANOVA followed by Tukey’s *post hoc* test for parametric data, or Kruskal–Wallis followed by Dunn’s *post hoc* test for nonparametric data (**p ≤ 0.01 and ****p ≤ 0.0001). For comparisons between culture systems, data were log-transformed to meet parametric assumptions and analyzed using two-way ANOVA, where “a” indicates statistically significant differences between cocultures and macrophage monocultures, and “b” indicates differences between cocultures and chondrocyte monocultures. The dotted line represents the limit of detection for each analyte.

To complement gene expression analyses and determine how individual and combined cellular contexts shape mediator release, cytokine, chemokine, and metalloproteinase concentrations were quantified in supernatants from macrophage monocultures, chondrocyte monocultures, and macrophage-chondrocyte cocultures following PsE stimulation (**Figure 4A**). Analysis of cytokine levels in culture supernatants revealed distinct production profiles depending on the cell culture system. In macrophage monocultures, PsE treatment resulted in a significant increase in IL-1β, IL-6 and TNF-α concentrations at 24 and 48 h compared to buffer-treated controls (**Figures 4B, F, J**), whereas IL-11 and IL-23 levels remained low or undetectable (**Figures 5A, E**). Chondrocyte monocultures showed only IL-6 production following PsE stimulation (**Figure 4G**), while the release of IL-1β, TNF-α (**Figure 4C** and K), IL-11 and IL-23 (**Figures 5B, F**) where under the detection limit. In contrast, macrophage–chondrocyte cocultures exhibited substantially higher concentrations of IL-6 upon PsE treatment, particularly at 48 h, compared with both buffer-treated cocultures (**Figure 4H**). This increase was consistently more pronounced in coculture than in either macrophage or chondrocyte monocultures (**Figure 4I**). While IL-11 and IL-23 were undetectable in macrophage and chondrocyte monocultures, their release was significantly increased exclusively under macrophage–chondrocyte coculture conditions following PsE stimulation (**Figures 5C, G**). By contrast, IL-1β and TNF-α concentrations were significantly lower in cocultures compared with macrophage monocultures (**Figures 4D, E, L, M**).

**Figure 6:**
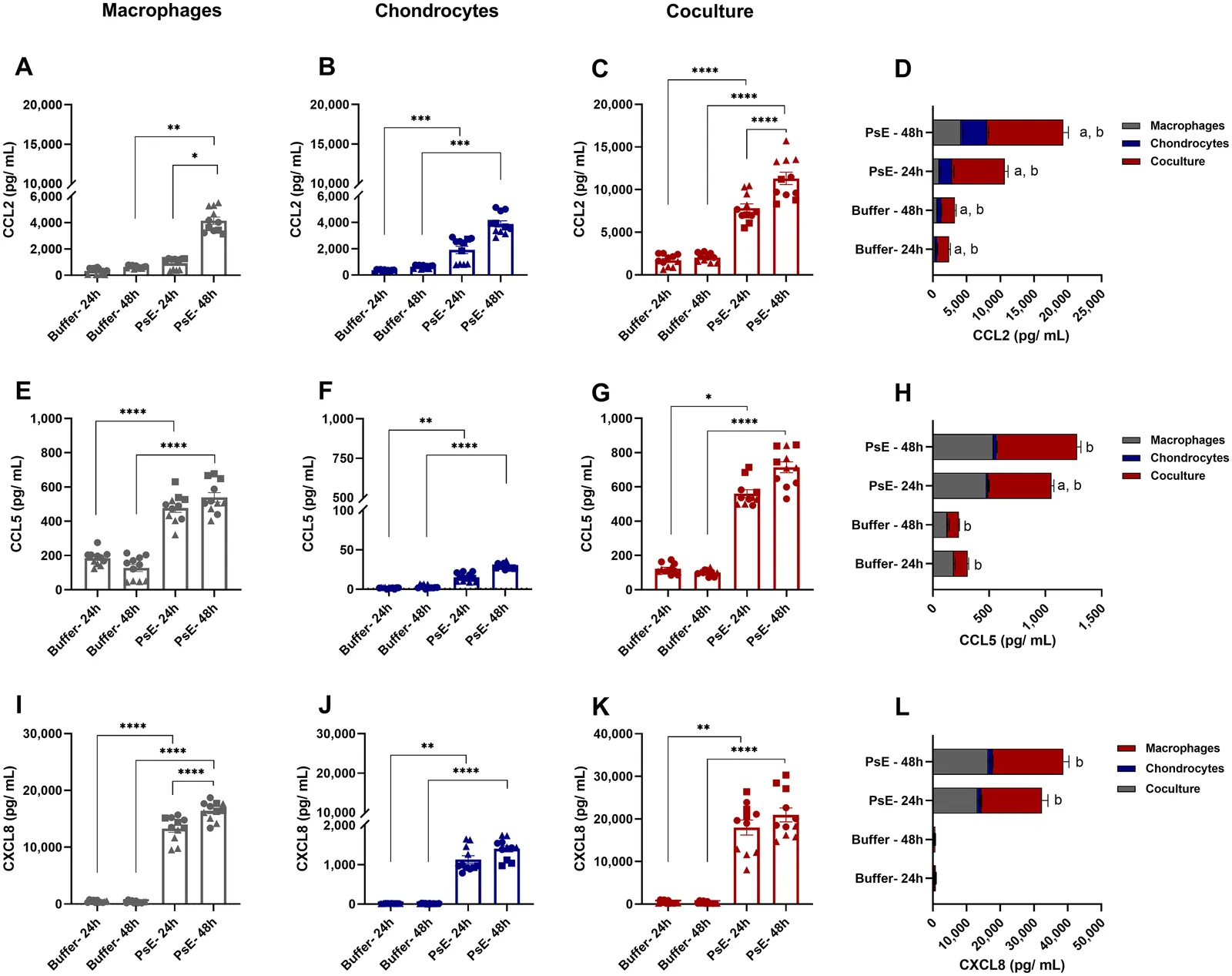
PsE modulates chemokines release in macrophage and chondrocyte cultures. THP-1–derived macrophages, chondrocytes, and macrophage–chondrocyte cocultures were stimulated with *P. semirufa* bristle extract (PsE; 60 µg/mL) or buffer (control) for 24 and 48 h **(A)**. The concentrations of CCL2 **(A-D)**, CCL5 **(E-H)**, and CXCL8 **(I-L)** in culture supernatants were quantified by cytometric bead array (CBA). Data are presented as dot plots showing the mean ± SEM of biological replicates from three independent experiments. Each data point represents an individual biological replicate, and symbol shape identifies the independent experiment (circles, Experiment A; triangles, Experiment B; squares, Experiment C). Data were assessed for normality prior to statistical analysis. Statistical analyses were performed within each culture system using one-way ANOVA followed by Tukey’s *post hoc* test for parametric data, or Kruskal–Wallis followed by Dunn’s *post hoc* test for nonparametric data (*p ≤ 0.05, **p ≤ 0.01, ***p ≤ 0.001 and ****p ≤ 0.0001). For comparisons between culture systems, data were log-transformed to meet parametric assumptions and analyzed using two-way ANOVA, where “a” indicates statistically significant differences between cocultures and macrophage monocultures, and “b” indicates differences between cocultures and chondrocyte monocultures.

The production of chemokines was also significantly affected by coculture conditions. In macrophage monocultures, PsE treatment increased the secretion of CCL2, CCL5, and CXCL8 at both time points compared to buffer controls (**Figures 6A, E, I**). Similar to chondrocyte monocultures that displayed moderate increases in chemokine levels following PsE exposure (**Figures 6B, F, J**). Notably, macrophage-chondrocyte cocultures showed markedly elevated concentrations of CCL2 and CCL5, following PsE treatment, with significantly higher levels of CCL2 observed at 48 h compared to 24 h (**Figures 6C, G**) and relative to both monoculture conditions (**Figures 6D, H**). CXCL8 concentrations were high in cocultures following PsE treatment (**Figure 6K**) and were comparable to macrophage monocultures, while remaining significantly higher than those observed in PsE-treated chondrocytes (**Figure 6L**). Buffer-treated cocultures showed minimal chemokine production, indicating that increased secretion was associated with PsE stimulation.

**Figure 7:**
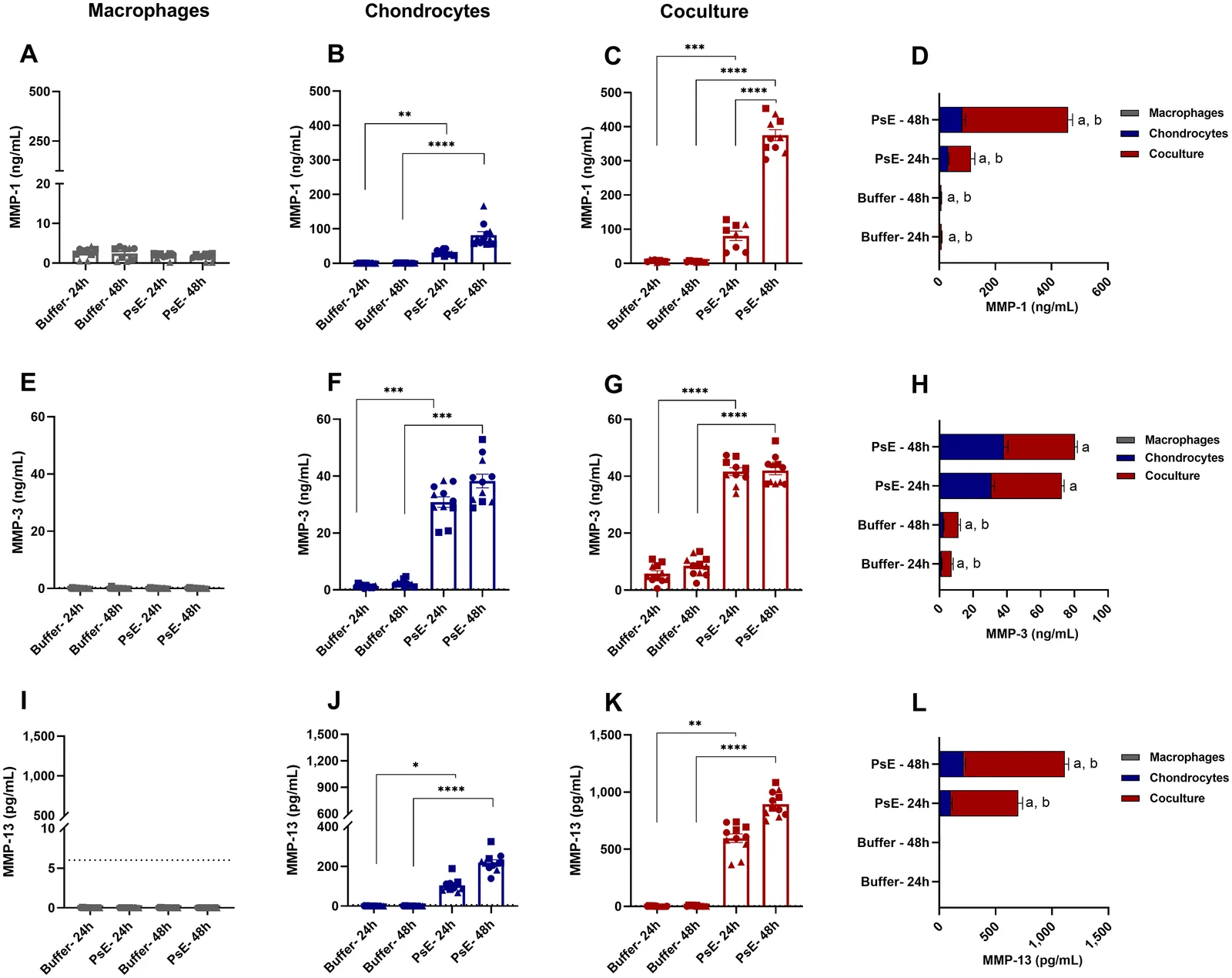
Macrophage–chondrocyte crosstalk enhanced the production of metalloproteinases in PsE-stimulated cultures. THP-1–derived macrophages, chondrocytes, and macrophage–chondrocyte cocultures were stimulated with *P. semirufa* bristle extract (PsE; 60 µg/mL) or buffer (control) for 24 and 48 h. The concentrations of MMP-1 **(A–D)**, MMP-3 **(E–H)** and MMP-13 **(I–L)** in culture supernatants were quantified by ELISA. Data are presented as dot plots showing the mean ± SEM of biological replicates from three independent experiments. Each data point represents an individual biological replicate, and symbol shape identifies the independent experiment (circles, Experiment A; triangles, Experiment B; squares, Experiment C). Data were assessed for normality prior to statistical analysis. Statistical analyses were performed within each culture system using one-way ANOVA followed by Tukey’s *post hoc* test for parametric data, or Kruskal–Wallis followed by Dunn’s *post hoc* test for nonparametric data (*p ≤ 0.05, **p ≤ 0.01, ***p ≤ 0.001 and ****p ≤ 0.0001). For comparisons between culture systems, data were log-transformed to meet parametric assumptions and analyzed using two-way ANOVA, where “a” indicates statistically significant differences between cocultures and macrophage monocultures, and “b” indicates differences between cocultures and chondrocyte monocultures. The dotted line represents the limit of detection for each analyte.

Quantification of matrix metalloproteinases in culture supernatants also demonstrated differential regulation across the culture systems. In macrophage monocultures, MMP-1, MMP-3, and MMP-13 levels were not detected (**Figures 7A, E, I**), while chondrocyte monocultures exhibited a high production of these MMPs after PsE exposure (**Figures 7B, F, J**). In macrophage–chondrocyte cocultures, PsE treatment led to a pronounced increase in the secretion of MMP-1, MMP-3, and MMP-13 (**Figures 7C, G, K**), with concentrations significantly higher than those observed in either monoculture condition (**Figures 7D, H, L**). These effects were more evident at 48 h particularly for MMP-1 and MMP-13, indicating a time-dependent accumulation of metalloproteinases in the coculture supernatants. Importantly, these changes in inflammatory mediators and metalloproteinase secretion were not associated with cytotoxic effects, as the viability of both monocultures and cocultures was only minimally affected by PsE treatment (**Supplementary Figure 1**).

### 3.4 IL-1β neutralization modulates cytokine and metalloproteinase release in macrophage–chondrocyte cocultures

**Figure 8:**
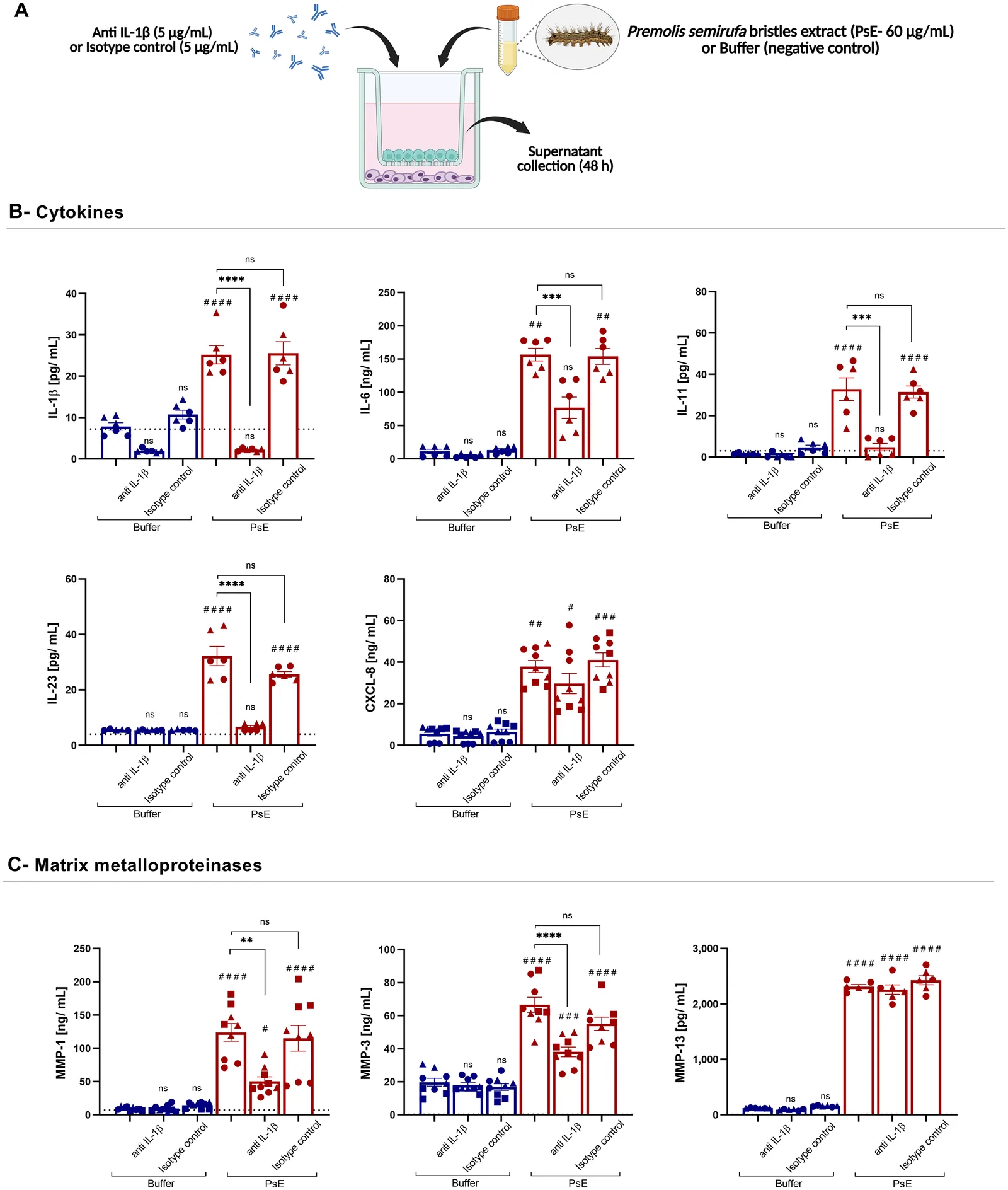
PsE modulates cytokine release in macrophage and chondrocyte cultures. THP-1 derived macrophages, chondrocytes, and macrophage–chondrocyte cocultures were stimulated with *P. semirufa* bristle extract (PsE; 60 µg/mL) or buffer (control) for 24 and 48 h **(A)**. The concentrations of IL-1β **(B–E)**, IL-6 **(F–I)**, and TNF-α **(J–M)** in culture supernatants were quantified by cytometric bead array (CBA). Data are presented as dot plots showing the mean ± SEM of biological replicates from three independent experiments. Each data point represents an individual biological replicate, and symbol shape identifies the independent experiment (circles, Experiment A; triangles, Experiment B; squares, Experiment C). Data were assessed for normality prior to statistical analysis. Statistical analyses were performed within each culture system using one-way ANOVA followed by Tukey’s *post hoc* test for parametric data, or Kruskal–Wallis followed by Dunn’s *post hoc* test for nonparametric data (** p ≤ 0.01, *** p ≤ 0.001 and **** p ≤ 0.0001). For comparisons between culture systems, data were log-transformed to meet parametric assumptions and analyzed using two-way ANOVA, where “a” indicates statistically significant differences between cocultures and macrophage monocultures, and “b” indicates differences between cocultures and chondrocyte monocultures. The dotted line represents the limit of detection for each analyte.

To investigate the contribution of IL-1β to the inflammatory and catabolic profile observed in macrophage–chondrocyte cocultures following PsE stimulation, an IL-1β neutralization assay was performed (**Figure 8A**). Neutralization of IL-1β did not significantly affect basal cytokine or metalloproteinase levels under buffer-treated conditions (**Figures 8B, C**). However, under PsE stimulation, IL-1β neutralization markedly altered cytokines and matrix metalloproteinases release in cocultures. In PsE-treated cocultures, IL-1β neutralization led to a significant reduction in cytokine concentrations compared with positive control or isotype-treated cells. Specifically, IL-1β, IL-11, and IL-23 levels were markedly decreased, reaching values comparable to those observed in cells not exposed to PsE. IL-6 levels were also significantly reduced following IL-1β neutralization in PsE-treated cells, although they remained elevated relative to the negative control, while CXCL8 was not significantly modulated (**Figure 8B**). Analysis of matrix metalloproteinases revealed a selective effect of IL-1β neutralization. While MMP-13 concentrations remained unchanged, neutralization of IL-1β significantly reduced the secretion of MMP-1 and MMP-3 in PsE-stimulated cocultures compared with isotype controls (**Figure 8C**).

## 4 Discussion

Pararamosis is an occupational osteoarticular condition triggered by the accidental penetration of human skin by the bristles of *Premolis semirufa*, a caterpillar endemic to the Amazon region (1, 6). Repeated exposure, particularly among rubber tappers, leads to a chronic inflammatory process that progressively compromises joint integrity, culminating in cartilage damage and structural deformities (1, 3). Despite advances in its characterization, therapeutic approaches for Pararamosis remain largely symptomatic. A deeper understanding of the molecular mechanisms driving this condition is therefore essential to elucidate its pathogenesis and to identify key inflammatory pathways that may represent potential therapeutic targets.

The present study demonstrates that *P. semirufa* bristle extract (PsE) acts as a potent activator of innate immune pathways in macrophages and promotes a coordinated inflammatory and catabolic response in macrophage-chondrocyte cocultures. Our findings demonstrate that IL-1β is a key mediator of macrophage-chondrocyte crosstalk, coordinating an inflammatory and catabolic response that shares important mechanistic features with other degenerative joint conditions, including osteoarthritis.

Macrophages are central orchestrators of synovial inflammation in osteoarticular diseases, with their functional polarization critically shaping disease outcomes (18, 21). Classically activated (M1-like) macrophages, characterized by high expression of CD80, CD86, and toll-like receptors (TLRs), produce pro-inflammatory mediators such as IL-1β, IL-6, TNF-α, and chemokines including CCL2 and CCL5, thereby amplifying leukocyte recruitment and sustaining inflammation (22, 23). These cytokines and chemokines promote inflammatory amplification and directly influence chondrocyte metabolism, reducing extracellular matrix (ECM) synthesis and increasing catabolic activity. A key mechanism underlying this response involves activation of the NLRP3 inflammasome, which promotes IL-1β maturation dependent on caspase-1 activity (24). This cytokine plays a pivotal role in joint pathology by suppressing ECM synthesis and enhancing the production of other inflammatory cytokines and matrix-degrading enzymes, ultimately contributing to inflammatory reactions and cartilage breakdown (25). Thus, pathways that regulate IL-1β production are central to the maintenance of chronic inflammatory processes in joint diseases.

In this context, our findings demonstrate that PsE induces a robust pro-inflammatory activation of macrophages, consistent with an M1-like phenotype, as evidenced by increased expression of CD14, CD80, CD86, and TLR2 (**Figure 2**). This response is accompanied by marked upregulation of *TLR2* and *TLR4*, together with increased expression of *NLRP3* and *CASP1* (**Figure 1**), indicating activation of both membrane-associated pattern-recognition receptor (PRR) pathways and inflammasome-related mechanisms in macrophages. In turn, activated macrophages initiated intracellular events that induced significant increase in the expression of important inflammatory cytokines (*IL1A*, *IL1B*, *IL-6*, *IL23A* and *IL33*), regulatory cytokines (*IL10*), chemokines (*IL8*) and growth factors (*VEGFA*, *TGFB1*) (**Figure 1**), as well as increased secretion of cytokines and chemokines in cell supernatant (**Figures 4**, **6**). Together, these data suggest that PsE acts as a damage-associated stimulus capable of engaging innate immune sensors and priming macrophages for sustained inflammatory amplification.

Although increased expression of inflammasome-related genes and elevated IL-1β secretion support the involvement of inflammasome-associated pathways, direct assessment of inflammasome assembly and caspase-1 cleavage was not performed and therefore warrants further investigation.

Notably, although macrophages displayed a strong inflammatory response to PsE, the magnitude and complexity of the response were significantly enhanced in the presence of chondrocytes. Chondrocytes, the sole resident cells of articular cartilage, are responsible for maintaining ECM homeostasis under physiological conditions. However, under inflammatory stress, they undergo phenotypic changes, including the upregulation of catabolic enzymes and inflammatory cytokines (26, 27).

In our study, chondrocytes cultured alone responded to PsE with a modest increase in inflammatory mediators and matrix-degrading enzymes expression and reduced expression of extracellular matrix components such as *COL2A* and *ACAN*. In contrast, in coculture with macrophages, these cells exhibited a markedly amplified response, including increased expression of *MMP3*, *MMP13*, as well as *IL1B* and *IL11*, which expression was detected only in chondrocytes exposed to co-cultured conditions (**Figure 3**). Consistent with these transcriptional changes, analysis of culture supernatants revealed that PsE stimulation increased the release of inflammatory mediators and metalloproteinases by chondrocytes, including IL-6, CCL2, CCL5, CXCL8, MMP-1, MMP-3, and MMP-13. This pattern is in agreement with previous studies showing that chondrocytes exposed to PsE adopt a catabolic and pro-inflammatory phenotype (12). Notably, the levels of several of these mediators were significantly higher in chondrocyte-macrophage cocultures (**Figures 4**-**7**), reinforcing the idea that cellular crosstalk enhances the inflammatory and catabolic response.

In particular, the elevated production of chemokines such as CCL2 and CCL5 in coculture conditions (**Figures 6D, H**) supports the establishment of a microenvironment favorable to immune cell recruitment. These mediators are known to regulate monocyte and lymphocyte trafficking and may play a role in sustaining local inflammation. In agreement with this, increased levels of CCL2 and CCL5 have been reported in the synovial fluid of individuals with joint injury and osteoarthritis, where they correlate with disease severity and immune cell infiltration (28–32). Moreover, previous *in vivo* findings demonstrated the infiltration of neutrophils and macrophages into the joints of mice inoculated with PsE, along with lymphocytes activation (7), further highlighting the relevance of immune cell and joint cell interactions in pararamosis.

Within this context of enhanced inflammatory signaling, we next examined the secretion of matrix metalloproteinases (MMPs), key effectors of ECM degradation. Although MMPs are essential for physiological tissue remodeling, their dysregulated production is a hallmark of joint pathology, contributing to cartilage breakdown (33, 34). While PsE stimulation induced the production of MMP-1, MMP-3, and MMP-13 in chondrocyte monocultures, these enzymes were not detected in macrophage monocultures. Importantly, coculture with macrophages led to a pronounced and time-dependent increase in MMP secretion, particularly for MMP-1 and MMP-13 (**Figure 7**), further supporting the role of intercellular crosstalk in amplifying cartilage-degrading mechanisms. The coculture system also revealed qualitative changes in the inflammatory profile that were not observed in monocultures. Importantly, IL-1β and TNF-α were detected in both macrophage monocultures and macrophage-chondrocyte cocultures but were absent in chondrocyte monocultures (**Figure 4**), supporting the idea that macrophages are the primary source of these cytokines within the joint environment.

These observations are in line with previous studies investigating cellular communication within the joint microenvironment. Transcriptomic analyses at the single-cell level have shown that pro-inflammatory cytokines such as IL-1β and TNF-α are mainly produced by immune cells, including macrophages and dendritic cells (35). Conversely, single-cell mass cytometry identified chondrocyte subpopulations with distinct functional profiles, including an inflammation-amplifying subset characterized by the expression of interleukin-1 receptor 1 (IL1R1) and tumor necrosis factor receptor II (TNFRII). Interestingly, inhibition of this subpopulation resulted in reduced inflammatory responses, suggesting that chondrocytes are not merely passive responders but can actively contribute to the amplification of inflammation (36).

In this context, the exclusive detection of IL-11 and IL-23 in cocultures following PsE stimulation (**Figures 5C, G**) supports that these pathways arise from macrophage–chondrocyte crosstalk rather than isolated cell activation, highlighting the role of coordinated intercellular signaling in shaping the joint inflammatory response. IL-11 is a multifunctional cytokine of the IL-6 family that, although minimally expressed under physiological conditions, has been increasingly associated with disease pathogenesis. It plays a key role in fibrosis across multiple organs by regulating cell proliferation, migration, differentiation, and angiogenesis (37–39), and has emerged as a potential therapeutic target currently under clinical investigation (40–42). Beyond its role in fibrotic disorders, elevated levels of IL-11 have been reported in the serum and synovial fluid of osteoarthritis patients (43). In addition, genetic and transcriptomic evidence further reinforces its relevance: a missense variant in the *IL11* gene has been associated with an increased risk of hip osteoarthritis, and *IL11* expression has been shown to be elevated in degraded cartilage compared to preserved tissue (44). Despite these advances, the precise role of IL-11 in cartilage homeostasis and degeneration remains incompletely defined.

In addition, IL-23 is a pro-inflammatory cytokine composed of two subunits, IL-23A (p19) and IL-12/23B (p40), the latter shared with Interleukin-12 (IL-12). IL-23 is mainly produced by macrophages and dendritic cells, in response to exogenous or endogenous signals, and drives the differentiation and activation of T helper 17 (Th17) cells with subsequent production of IL-17 and other pro-inflammatory mediators (45). Extensive knowledge supports the contribution of its dysregulation in triggering chronic inflammation and autoimmunity, including psoriatic arthritis, rheumatoid arthritis and osteoarthritis (46–48). Supporting this axis, a murine model of pararamosis demonstrated increased levels of IL-23 and IL-17, along with the expansion of Th17 lymphocytes following repeated PsE administration, consistent with the establishment of a sustained inflammatory response (7). Although T cells were not directly evaluated in this study, the increased expression and secretion of IL-23A in macrophage–chondrocyte cocultures suggest that PsE exposure may create a microenvironment favorable to Th17 polarization. In addition, the concomitant increase in IL-1β and IL-6, both recognized as critical factors in Th17 differentiation, supports the notion that this cytokine network may act synergistically to sustain inflammatory responses.

A central finding of this study is the identification of IL-1β as a critical upstream regulator of macrophage–chondrocyte communication. Neutralization experiments revealed that IL-1β blockade significantly reduced IL-1β, IL-6, IL-11, IL-23, MMP-1, and MMP-3 levels in cocultures (**Figures 8B, C**), demonstrating that IL-1β, within a multicellular context, functions not merely as an inflammatory product but as a hierarchical regulator of downstream cytokine and catabolic mediator production. Interestingly, CXCL8 and MMP-13 levels remained unaffected by IL-1β neutralization (**Figures 8B, C**), suggesting the coexistence of IL-1β-dependent and IL-1β-independent pathways. This observation aligns with evidence that CXCL8 and MMP-13 expression can be driven by alternative signaling axes in chondrocytes (49), for example through MAPK activation induced by IL-6 and TNF-α, cytokines also upregulated in macrophage-chondrocyte cocultures under PsE stimulus. These findings indicate that IL-1β plays a major regulatory role in the inflammatory network induced by PsE, while also highlighting the contribution of additional signaling pathways that may act independently or in parallel.

Although IL-1β neutralization provided functional evidence supporting its contribution to the inflammatory response, the intracellular mechanisms underlying these effects remain to be elucidated. In particular, the involvement of signaling pathways such as NF-κB, MAPK, and other downstream regulatory networks was not directly investigated in the present study and remains to be determined.

Notably, IL-1β concentrations were lower in cocultures compared to macrophage monocultures (**Figure 4E**), despite its central role in driving the inflammatory response. This finding may reflect dynamic regulatory mechanisms within the coculture system, such as increased cytokine consumption, receptor engagement, or feedback inhibition. More importantly, it highlights that the biological relevance of a cytokine within a multicellular inflammatory network is not necessarily reflected by its absolute extracellular concentration. Cytokines acting upstream in signaling cascades may exert profound regulatory effects even at relatively low concentrations by triggering the production of multiple downstream mediators. Consistent with this concept, IL-1β neutralization markedly reduced the secretion of IL-11, IL-23, MMP-1, and MMP-3, supporting its role as a key upstream regulator of the inflammatory and catabolic response induced by PsE. These findings identify IL-1β as an important mediator of macrophage–chondrocyte crosstalk and provide a rationale for further studies investigating its contribution to disease progression *in vivo*.

Corroborating, our previous report demonstrated the infiltration of neutrophils and macrophages into the joints of mice inoculated with PsE (7), reinforcing the importance of immune cells interactions with joint cells in pararamosis. Our data suggest that PsE initiates an IL-1β–dependent response that may sustain inflammatory signaling loops within the joint microenvironment, contributing to prolonged innate immune activation and recruitment of additional inflammatory populations via chemokine induction.

## 5 Conclusion

**Figure 9:**
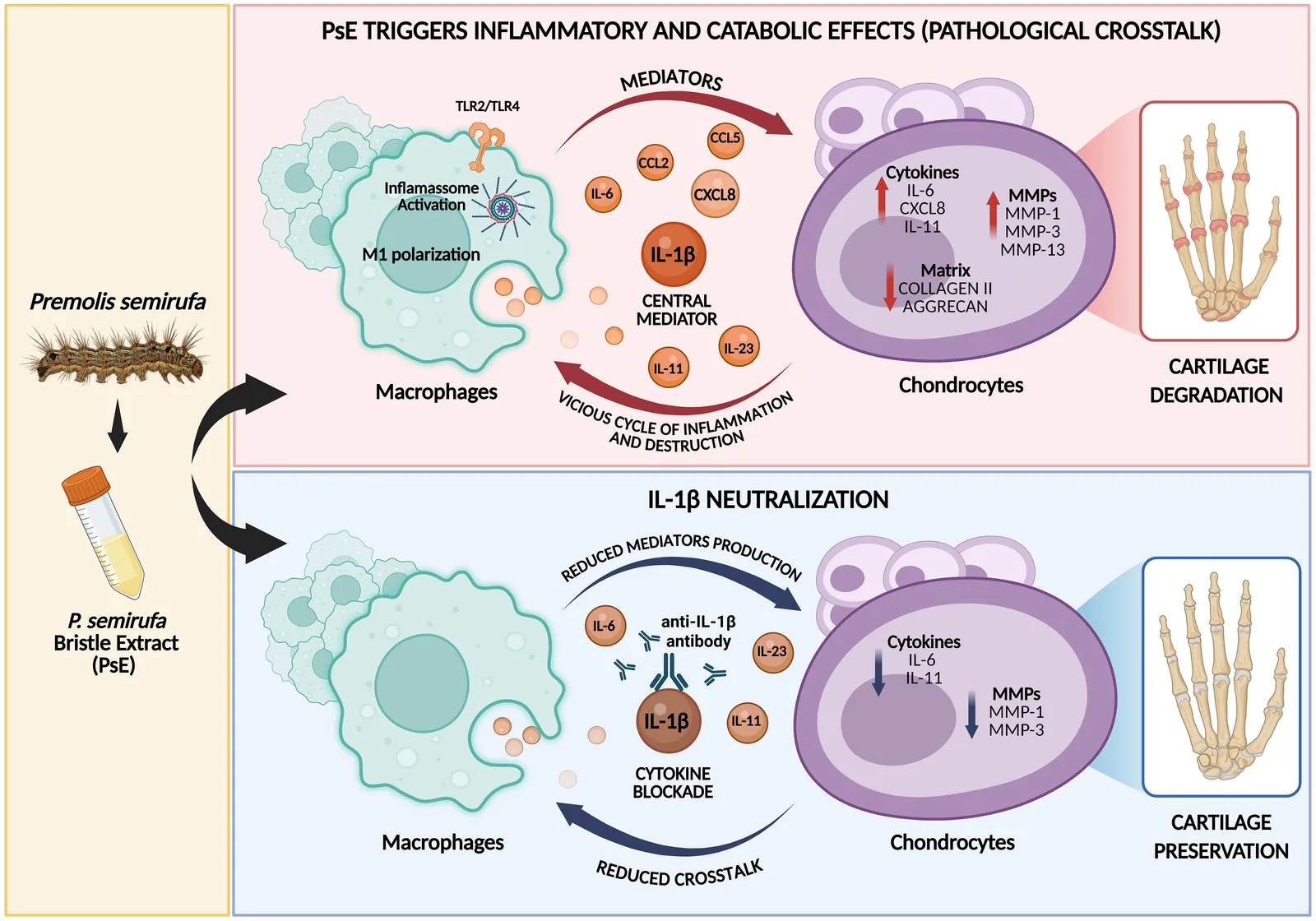
Proposed model of PsE-induced inflammatory and catabolic crosstalk in pararamosis and the effects of IL-1β neutralization. Exposure to *Premolis semirufa* bristle extract (PsE) promotes macrophage activation through TLR2/TLR4 signaling, inflammasome activation, and M1 polarization, resulting in the release of inflammatory mediators, particularly IL-1β, together with IL-6, CCL2, CCL5, and CXCL8. IL-1β acts as an important mediator amplifying macrophage–chondrocyte crosstalk and stimulating chondrocytes to produce inflammatory cytokines (IL-6, CXCL8, and IL-11) and matrix-degrading enzymes (MMP-1, MMP-3, and MMP-13), while reducing extracellular matrix components such as collagen II and aggrecan. The induction of IL-11 and IL-23 may further contribute to chronic inflammation and tissue remodeling, sustaining a vicious cycle of inflammation and cartilage destruction. In contrast, IL-1β neutralization attenuates mediator production, reduces macrophage–chondrocyte crosstalk, decreases cytokine and MMP expression, and contributes to cartilage preservation, highlighting IL-1β as a potential therapeutic target in pararamosis-associated joint pathology. Created in https://BioRender.com .

Collectively, our findings support a model in which PsE acts as a potent trigger of innate immune activation, initiating an inflammatory signaling network in which IL-1β plays an important regulatory role and that is further amplified through macrophage-chondrocyte crosstalk. This interaction not only enhances the production of inflammatory mediators and matrix-degrading enzymes but also promotes the emergence of additional regulatory pathways, including IL-11 and IL-23, which may contribute to tissue remodeling and the establishment of a sustained inflammatory milieu. These results highlight that joint damage in pararamosis is driven by dynamic intercellular interactions rather than isolated cellular responses. Targeting key nodes within this network, particularly IL-1β and related pathways, may represent a promising strategy to limit chronic inflammation and cartilage degradation. A schematic model summarizing the proposed PsE-induced inflammatory and catabolic network, including macrophage–chondrocyte crosstalk and downstream mediators involved in cartilage damage, is presented in **Figure 9**.

## Data Availability

The raw data supporting the conclusions of this article will be made available by the authors, without undue reservation.
