# Supplementary material for: IL-1β as an important mediator of macrophage–chondrocyte crosstalk in pararamosis (rainforest arthritis)
Source: Front Immunol. 2026 Aug 25;17:1880784. doi: 10.3389/fimmu.2026.1880784 (PMC13549860; doi:10.3389/fimmu.2026.1880784)
Supplement: Supplementary file 1 [file SupplementaryFile1.docx]

**Figure S1.** **Viability of macrophages and chondrocytes after PsE treatment**. THP-1 derived macrophages and chondrocytes, cultured either as monocultures or cocultures, were stimulated with *Premolis semirufa* bristle extract (PsE; 60 µg/mL) or buffer (control) for 48 h. Cell viability was assessed by MTT assay and expressed as a percentage relative to control. Data are presented as mean ± SEM. Statistical analysis was performed using Student’s t-test comparing PsE-treated cells to their respective buffer controls (p ≤ 0.05, *p ≤ 0.01, **p ≤ 0.001).
