# Supplementary material for: IL-1β as an important mediator of macrophage–chondrocyte crosstalk in pararamosis (rainforest arthritis)
Source: Front Immunol. 2026 Aug 25;17:1880784. doi: 10.3389/fimmu.2026.1880784 (PMC13549860; doi:10.3389/fimmu.2026.1880784)
Supplement: Supplementary file 2 [file Table1.docx]

**Table S1. List of primers**

| Gene | GenBank acession number | Primer sequence |
| --- | --- | --- |
| *ACAN* | NM_001369268.1 | Fw: AAGACGGCTTCCACCAGTGT |
|  |  | Rv: ATGCCATACGTCCTCACACC |
| *ASC* | NM_013258.5 | Fw: CAAACGTTGAGTGGCTGCTG |
|  |  | Rv: ATCTTGCTTGGGTTGGTGGG |
| *CASP1* | NM_033292.4 | Fw: TGCCTGTTCCTGTGATGTGG |
|  |  | Rv: GCATCTGCGCTCTACCATCT |
| *COL2A1* | NM_001844.5 | Fw: CCAGATGACCTTCCTACGCC |
|  |  | Rv: TCAGGGCAGTGTACGTGAAC |
| *IL1A* | NM_000575.5 | Fw: CAGCCAGAGAGGGAGTCATTT |
|  |  | Rv: GTCTGGAACTTTGGCCATCTTG |
| *IL1B* | NM_000576.3 | Fw: GTACCTGTCCTGCGTGTTGA |
|  |  | Rv: GGGAACTGGGCAGACTCAAA |
| *IL6* | NM_000600.5 | Fw: GTACATCCTCGACGGCATCTC |
|  |  | Rv: TCACCAGGCAAGTCTCCTCAT |
| *IL8* | NM_000584.4 | Fw: CACCGGAAGGAACCATCTCAC |
|  |  | Rv: GGCAAAACTGCACCTTCACAC |
| *IL10* | NM_000572.3 | Fw: GCTGAGAACCAAGACCCAGACA |
|  |  | Rv: TAAAGGCATTCTTCACCTGCTCC |
| *IL11* | NM_000641.4 | Fw: GCTGCACCTGACACTTGACT |
|  |  | Rv: ATCTGGCTTTGGAAGGACGG |
| *IL18* | NM_001562.4 | Fw: AGCTGAAGATGATGAAAACCTGG |
|  |  | Rv: ATATGGTCCGGGGTGCATTA |
| *MMP1* | NM_002421.4 | Fw: CACGCCAGATTTGCCAAGAG |
|  |  | Rv: TTGTCCCGATGATCTCCCCT |
| *MMP3* | NM_002422.5 | Fw: CACTCACAGACCTGACTCGG |
|  |  | Rv: AAGCAGGATCACAGTTGGCT |
| *MMP13* | NM_002427.4 | Fw: GCCATTACCAGTCTCCGAGG |
|  |  | Rv: TACGGTTGGGAAGTTCTGGC |
| *NLRP3* | NM_004895.5 | Fw: CTCCCAAGCTCCTCTCATGC |
|  |  | Rv: AGAACTGAAAAGAGGCCCCG |
| *TGFB1* | NM_000660.7 | Fw: TCCTGGCGATACCTCAGCAA |
|  |  | Rv: CTCAATTTCCCCTCCACGGC |
| *TNC* | NM_002160.4 | Fw: TCAAAGACGTGCCAGGAGAC |
|  |  | Rv: CTTTGGCTGGGTTGCTTGAC |
| *TLR2* | NM_003264.5 | Fw: TGTGGATGGTGTGGGTCTTG |
|  |  | Rv: TGCAGATACCATTGCGGTCA |
| *TLR4* | NM_003263.4 | Fw: CAGGAACACTTACCTGGAGTGG |
|  |  | Rv: CTGTACCCACTGTTCCTTCTGG |
| *VEGFA* | NM_001025366.3 | Fw: CCCACTGAGGAGTCCAACATC |
|  |  | Rv: CTGCATTCACATTTGTTGTGCTG |
| *RPL13A* | NM_012423 | Fw: GTATGCTGCCCCACAAAACC |
|  |  | Rv: CTTCAGACGCACGACCTTGA |
| *PPIA* | NM_021130.5 | Fw: GCGTCTCCTTTGAGCTGTTT |
|  |  | Rv: CCCTGACACATAAACCCTGGA |
| *GAPDH* | NM_002046.7 | Fw: CCCACTCCTCCACCTTTGAC |
|  |  | Rv: CCACCACCCTGTTGCTGTAG |
